# Multicentre clinical evaluation of the safety and performance of a simple transperineal access system for prostate biopsies for suspected prostate cancer: The CAMbridge PROstate Biopsy DevicE (CamPROBE) study

**DOI:** 10.1177/2051415820932773

**Published:** 2020-06-12

**Authors:** Vincent J Gnanapragasam, Kelly Leonard, Michal Sut, Cristian Ilie, Jonathan Ord, Jacques Roux, Maria Consuelo Hart Prieto, Anne Warren, Priya Tamer

**Affiliations:** 1 Cambridge Urology Translational Research and Clinical Trials Office, Cambridge University Hospitals NHS Foundation Trust, United Kingdom; 2 Academic Urology Group, Department of Surgery, University of Cambridge, United Kingdom; 3 Department of Urology, Cambridge University Hospitals Trust, United Kingdom; 4 Department of Urology, North West Anglia NHS Trust, United Kingdom; 5 Department of Urology, The Queen Elizabeth Hospital Foundation Trust, United Kingdom; 6 Department of Urology, Cheltenham and Gloucester Hospital, United Kingdom; 7 Department of Urology, West Hertfordshire Hospitals NHS Trust, United Kingdom; 8 Department of Urology, Aneurin Bevin Hospital, United Kingdom; 9 Department of Pathology, Cambridge University Hospitals NHS Foundation Trust, United Kingdom

**Keywords:** CamPROBE, local anaesthetic, prostate cancer, transrectal biopsy, transperineal biopsy, safety, infection, clinical evaluation, first in man, NIHR

## Abstract

**Objectives:**

To report the prospective multicentre clinical evaluation of a first-in-man disposable device, Cambridge Prostate Biopsy Device, to undertake local anaesthetic outpatient transperineal prostate biopsies.

**Material and methods:**

Disposable single-use Cambridge Prostate Biopsy devices were manufactured based on a previous prototype. The lead site developed a user training course and disseminated the method to other sites. The Cambridge Prostate Biopsy Device (CamPROBE) was offered as an alternative to transrectal ultrasound guided biopsy to men due for a biopsy as part of their clinical management. Data on safety (infections and device performance), clinical utility, patient reported experience, biopsy quality and cancer detection were collected. Procedure time and local anaesthetic use was recorded in the lead site. The study was funded by a United Kingdom National Institute for Health Research (NIHR) i4i product development award.

**Results:**

A total of 40 patients were recruited (median age 69 y) across six sites; five sites were new to the procedure. Overall, 19/40 were first prostate biopsies and 21/40 repeat procedures. Both image-targeted and systematic biopsy cores taken. There were no infections, device deficiencies or safety issues reported. The procedure was well tolerated with excellent patient-reported perception and low pain scores (median of 3, scale 0–10). Histopathology quality was good and the overall cancer diagnosis rate (first diagnostic procedures) was 68% (13/19) and for significant cancers (⩾ histological Grade Group 2), 47% (9/19). In the lead centre (most experienced), median procedure time was 25 minutes, and median local anaesthetic use 11 ml (*n*=17).

**Conclusions:**

Data from this device evaluation study demonstrate that the United Kingdom-developed Cambridge Prostate Biopsy Device/method for transperineal biopsies is safe, transferable and maintains high diagnostic yields. The procedure is well tolerated by patients, suited to the local anaesthetic outpatient setting and could directly replace transrectal ultrasound guided biopsy.

**Level of evidence:**

Level III

## Introduction

Prostate cancer is the commonest male malignancy and its incidence is set to rise in the next few decades.^1^ Presently, the histological diagnosis of cancer is most commonly based on a transrectal ultrasound guided biopsy of the prostate (TRUSBx) under local anaesthesia (LA). This method is economical, facilitates image-guided targeting and is ideally suited to the outpatient setting as it requires low amounts of LA. As a result, more than 80% of prostate biopsies are performed this way, accounting for >1 million annual procedures in the western world alone.^2-4^ TRUSBx are, however, associated with a significant risk of biopsy associated infection (up to 22%) and sepsis (up to 10%) because the needle has to repeatedly traverse the rectal wall.^5-7^ Batura *et al*. modelled the resource impact of post-biopsy sepsis in the National Health Service and estimated an annual cost burden of £7–11 million.^8^ Another major concern is the increasing incidence of prostate biopsy-related antibiotic resistance. Carignan *et al.* (Canada) and Johansen *et al.* (Norway) have reported an up to four-fold increase in antibiotic resistant over the last decade alone and this experience is mirrored in many other countries.^7^,^9-12^ This is thus a major safety concern for health services globally every year. Transperineal (TP) biopsies are much less risky but more painful as the needles pass through the perineal skin and pelvic-floor musculature hence usually necessitating a general anaesthetic or sedation. As a result, there has been a concerted effort by many clinicians to explore how this can be done under LA. Current options, however, are costly and are still reported to need significant amounts of LA infiltration.^13-14^ There is thus an imperative need to eliminate the risk of infection inherent in TRUSBx while maintaining its simplicity, wider accessibility and low cost suitable for the routine outpatient setting.

To address this, we developed the CAMbridge PROstate Biopsy DevicE (CamPROBE) based on the concept of a co-axial cannula, but designed specifically for TP prostate biopsies under LA. The CamPROBE is inserted at two points on either side of the perineum mid-line. It is then advanced to the prostate with simultaneous targeted LA infiltration to deeper structures (including pelvic muscles) using the integrated delivery needle and under transrectal ultrasound guidance. Once in position, the needle is removed and the CamPROBE cannula can be used as an access sheath for prostate biopsies thus limiting tissue trauma and pain. The CamPROBE can be angled or repositioned to reach different areas without superficial or deep structure re-puncture. The device was first reported as a reusable stainless-steel prototype in a single-center study and showed excellent performance characteristics.^15^ Here we report the first-in-man evaluation of a new single-use disposable version of the CamPROBE device and its performance in a multi-centre stetting for its safety and performance as a potential direct alternative to TRUSBx.

## Methods

### Study cohort and outcome measures

**Figure 1.**
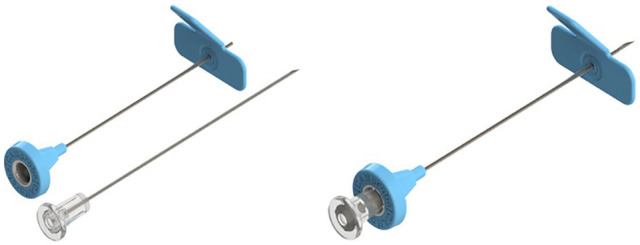
Images of the disposable CAMbridge PROstate Biopsy DevicE (CamPROBE) used in this clinical investigation. Its use can be seen here : https://www.youtube.com/watch?v=Q3XYLq5po8s&t=196s.

Disposable single-use CamPROBE devices (United Kingdom (UK) patent: P4256lGB) were manufactured and assembled within the Controlled Environment Manufacturing Assembly Facility at JEB Technologies Ltd of Mildenhall, Suffolk, UK. The study was funded by a UK National Institute of Health Research (NIHR) i4i grant for device development and a prospective multicentre clinical investigation (NCT0360952). The study was reviewed and received favourable ethical opinion by the East of England – Cambridge Central Ethics Committee (REC 18/EE/0272, IRAS Project ID: 242948). The lead site developed a user training course and disseminated the method to five other centres and this was supported by onsite mentoring. The CamPROBE device was offered as an alternative to TRUSBx to men due for a prostate biopsy as part of their standard clinical management. The device and step by step method can be viewed on youtube here: https://www.youtube.com/watch?v=Q3XYLq5po8s&t=196s (Figure 1). The primary outcome measure was safety as assessed by (a) the incidence of biopsy-related infection and (b) safety of the device in terms of device deficiencies. Data were also collected on clinician-reported device performances and patient experience using self-reported tools (composite discomfort and procedure perception scores and visual analogue scale for pain) (Supplementary Files 1 and 2). As this was a first-in-man study, the biopsy pattern was not standardised and left to the centres’ own standard of care. All centres did, however, use a combination of cognitive guided image-targeting (based on magnetic resonance imaging (MRI)) and systematic biopsies and the technical quality of the samples was assessed by an independent consultant histopathologist (AW). Cancer diagnosis rates were assessed using two criteria: (a) detection of any prostate cancer and (b) detection of disease of histological type Grade Group 2 or above (considered clinically significant disease) on the International Society of Uro-Pathology scale.^16^ The median procedure time and amount of LA used was recorded in the lead centre with the most experience of using the device.

### Statistical analysis

The *a priori* sample size was based on the primary outcome measure of reduced infections and derived as a minimum of 31 patients required to detect a reduced infection rate of ⩽ 1% with a power of 80% and at the 0.025 level of significance (versus reported prevalent UK rates for TRUSBx).^17^ We therefore aimed for a target group of 40 to allow for any dropouts. All clinical data produced from the CamPROBE study were collected and managed by the Cambridge Clinical Trials Unit – Cancer Theme. Data were entered on a secure MACRO electronic database, transcribed from paper CRFs (Case Report Forms) received from participating sites. Queries were raised in the validated, CCTU-CT generated, Trial Manager database to ensure a full audit trail for the data management processes. A full data quality assurance process was followed in line with the Sponsor’s standard operating procedures, whereby a Data Manager, independent of the trial team, tested for data entry transcription error rates of less than 2.5% in primary endpoint data points, and less than 5% in all other endpoint data points.

## Results

### Study population

In total 56 men were screened and 40 patients were recruited (median age 69 y) over an 8-month period (March–October 2019). All had successful biopsies across six centres. The indication was first diagnostic biopsies in 19/40 men and repeat procedures in 21/40 (men on cancer surveillance or negative on first biopsy). Both targeted (lesions defined on a prebiopsy prostate MRI) and systematic biopsy cores were taken (standard of care) as appropriate based on the centres own in-house protocol. In all cases two devices were used per patient (right and left sides) of the prostate (80 devices used in total). The total number of biopsy cores taken was 583 with a median of 12 samples per patient.

### Safety

Table 1 summarises the key outcomes from the investigation. All biopsies were completed successfully. The main objective of this investigation was to assess the safety of the CamPROBE device as a method of undertaking prostate biopsies. There were no reported infections related to the device or procedure within the 30-day follow-up after the CamPROBE procedure based on patient self-reported follow-up questionnaires (Table 1). Two patients did report fevers but neither event was related to evidence of a urinary tract or local biopsy site infection. There were also no device deficiencies or safety issues reported in any device use from any of the centres (0/80) (Table 1).

**Table 1.** Summary of results from the CamPROBE clinical investigation detailing the demographics and outcomes.

Patient group (*n*=40)	Results
Age (range)	69 years (49–79)
First biopsy procedure	19
Repeat biopsy procedure	21
**Procedure outcome (safety and infections) (*n*=80)** ^a^	
Biopsies completed	80/80 (100%)
Device failure/deficiencies	0/80 (0%)
Biopsy related infections/sepsis	0/80 (0%)
**Clinical performance (utility) (*n*=80)**	
Technical difficulty acquiring biopsies	6/80 (7.5%)^b^
Technical difficulty delaying or impeding biopsies	0/80 (0%)
**Procedure metrics – assessed at lead site only (*n*=17)**	
Median biopsy time (patient in to patient out) (range)	25 minutes (13–40)
Median local anaesthetic used (range)	10.5 ml (9–16)
**Patient reported outcomes (*n*=40)**	
Median discomfort score (scale 0–54)^c^ (range)	13 (2–42)
Median pain score (scale 0–10)^c^ (range)	3 (0–7)
Median overall perception (0–18)^c^ (range)	4 (0–14)

CamPROBE: CAMbridge PROstate Biopsy DevicE

a Each patient had two devices used for the right and left sides of the prostate.

b Reported on initial passage of the needles but subsequently resolved spontaneously.

c Higher score denotes higher discomfort, worse pain and worse perception.

### Clinical utility

Clinical performance was assessed for packaging integrity, need for replacements and device performance during biopsy taking. There was no issue with the receipt and packaging of the device and no need for any replacement devices. Clinicians reported very good functionality in the vast majority of cases. Once the CamPROBE integrated cannula is in place, standard biopsy needles are inserted into it to access the prostate and take samples (Figure 1). There were a few instances of some initial resistance to biopsy needle insertion into the CamPROBE cannula in 6/80 (7.5%) procedures. However, in all cases these resolved on subsequent biopsy needle passages (Table 1). None of these precluded successful completions of the procedure. A total of 17 procedures were performed in the lead centre, which had the greatest experience with using the device. Here the median procedure time was 25 minutes, and median amount of LA use was 11 ml (Table 1).

### Patient perception

Patient compliance rates were high in this investigation with no biopsy stopped prematurely. Patient reported outcomes measured on Day 1 post biopsy were good with low pain, discomfort and perception scores (Table 1). The overall median pain score was 3 for the whole procedure. Patients were also directly asked of their opinion of the CamPROBE biopsy. This was specifically relevant to men (*n*=21) who had already had a previous standard transrectal ultrasound guided biopsy (Table 2). The majority of respondents favoured the CamPROBE approach with a median score of 9/10. Similarly, most respondents would prefer the CamPROBE approach if they needed another biopsy again in the future (median score of 10) (Table 2). Over 85% would also recommend the CamPROBE to someone else as a method of having a prostate biopsy done.

**Table 2.** **Table detailing the scores for patient reported views on the CamPROBE.** Q1 score range 0 to 10 (0 being worse and 10 being better), Q2 score range 0 to 10 (0 less likely, 10 being more likely).

**Q1. How would you compare the CamPROBE biopsy with previous experience of standard transrectal ultrasound guided prostate biopsy?**	
Number of respondents	20
Mean (standard deviation)	8 (2)
Median (minimum, maximum)	9 (2, 10)
Inter-quartile range	6, 9
**Q2. If you had to have another prostate biopsy, are you more or less likely to want CamPROBE biopsy compared to normal transrectal biopsy?**	
Number of respondents	20
Mean (Standard deviation)	9 (2)
Median (minimum, maximum)	10 (3, 10)
Inter-quartile range	9, 10
**Q3. If you had a friend or relative who was about to have first prostate biopsy, which method would you recommend? *n/N* (%)**	
Standard transrectal biopsy	2/21 (9.5)
CamPROBE biopsy	18/21 (85.7)
Either	1/21 (4.8)

CamPROBE: CAMbridge PROstate Biopsy DevicE.

### Biopsy quality and cancer diagnosis rates

The technical quality of the biopsies was assessed by central pathology review. In 39/40 (98%) cases (representing use of 79/80 devices) this was sufficient for a clinical diagnosis and a management decision to be made. In one procedure, however, there was insufficient material from one side of the prostate, hence making an overall final histological diagnosis not possible. The independent histopathology reviewer confirmed that this could have happened with any type of prostate biopsy and was not specific to the CamPROBE device. We further assessed the cancer diagnosis rates in a subgroup analysis of men who were having their first biopsy for suspected prostate cancer (19/40) (Table 3). The overall cancer detection rate was 68% and for significant cancers, 47% (Table 3). These rates compare very favourably to either contemporary TRUSBx (48% and 35% respectively) or general anaesthetic grid-based MRI guided TP biopsy series (67% and 48% respectively, Table 3).

**Table 3.** Comparison of cancer detection rates between CamPROBE biopsies and other contemporary methods.

Biopsy type as first procedure	Number	Overall cancer detection rate (%)	Significant cancer detection rate (%)
Transrectal ultrasound guided biopsy^a^	714	344 (48.1)	256 (35.8)
General anaesthetic transperineal biopsies^b^	807	546 (67.6)	392 (48.5)
CamPROBE	19	13 (68.4)	9 (47.4)

CamPROBE: CAMbridge PROstate Biopsy DevicE.

a Composite data of 4 studies: Borkowetz et al. (2017),^18^ Baco et al. (2016),^19^ Porpiglia et al. (2016),^20^ Tonttila et al. (2016).^21^

b Hansen et al. (2016) multi-center study using template grid-based biopsies.^22^

## Discussion

The results from this study demonstrate that the CamPROBE device/method is safe and yields prostate biopsies to a similar standard to current methods. In keeping with the TP route, there were no infective or sepsis events following biopsy. In this investigation we did not identify any specific risks to using the CamPROBE versus any other prostate biopsy technique. The main risk is associated with the introduction of a new technique but appropriate training and certification would be the same for any new intervention method. All devices worked as expected with no device deficiencies and only minor issues reported in a few cases. This is particularly encouraging as the CamPROBE was new to five clinical teams in the investigation, suggesting the device and method can be readily disseminated and adopted. Training and credentialing are an essential part of any new procedure and in this regard, we have devised a training programme that was used at the start of this clinical investigation. No special precautions were needed beyond those common for any biopsy (e.g. stopping anticoagulants). Indeed, because of its demonstrably lower infective risks it may be a safer way of undertaking biopsies in men who are at high risk of infection (e.g. immunocompromised).

Histological quality was comparable to other standard means of prostate biopsy with only one in 40 cases where some cores were inadequate for conclusive analysis. This was benchmarked against nationally accepted standards and are within the accepted range of samples insufficient to diagnose cancer/needing a repeat biopsy. The British Association of Urological Surgeons quoted rates for sample inadequacy from TRUSBx, for example, is one in 50 (2%).^23^ Ubhayakar *et al.* (2002) have also previously reported poor-quality samples in up to 6% of TRUSBx-acquired biopsy cores.^24^ We therefore did not consider this a device-related issue, but one common to any prostate biopsy method. Cancer detection rates using the CamPROBE method also appeared to be on a par with contemporary published series regardless of definition used. We interpret this with caution as our cohort numbers were comparativley small. Nevertheless, it is encouraging that the data shows the CamPROBE method appears to be at least non-inferior to current biopsy methods. It is unlikely that any particular biopsy device and method is going to show superiority over another when standardised by numbers of cores taken and the use of MRI scans to guide targeting biopsies. Indeed, head-to-head comparisons of TRUSBx versus TP approaches in the pre-MRI era have shown no differences in cancer detection rates.^25, 26^ In the modern era the use of MRI has been shown to be a clear factor in increasing detection rates regardless of whether this is by TRUSBx or TP approaches.^22, 27-30^ Thus, we do not expect that the CamPROBE (or any other device) will be the gold standard or only way to carry out LA TP biopsies in the future. Instead any future optimal LA TP biopsy method will combine clinician skill, use of image guidance (cognitive or fusion), cost efficiency and procedure simplicity to fit into routine outpatient clinical practice. A key element is also patient acceptability. In this investigation, patient-reported feedback showed excellent results with low pain, discomfort and perception scores. Those who had previous experience with transrectal biopsies expressed an overwhelming preference for the CamPROBE method if they required a further biopsy in future. In context, Rosario et al. (2012) reported that one in five men would have a moderate/major problem with having a similar repeat procedure following their initial experience with a transrectal prostate biopsy.^17^ Initial data from the lead site also suggested LA volume used were low with experience and comparable to that used for TRUSBx. This observation, however, needs verification in larger future studies and by independent centre users.

In summary this evaluation study has shown that the CamPROBE is safe, reliably takes prostate biopsies under LA, can be easily clinically disseminated, is well received by patients and appears non-inferior in terms of cancer detection rates. Most importantly it achieves this with no apparent infective risks. The device cost is also projected to be low given its simple design and the low cost of materials. There are inevitable inherent limitations in this investigation namely, that it was a first-in-man evaluation and hence not a randomised study and included a relativley small sample size. Nevertheless, these results provide a sound basis on which to further develop and introduce the CamPROBE as a safer alternate biopsy method to TRUSBx into routine clinical practice. Future clinical investigation trial will aim at confirming the veracity of our findings, develop head to head comparisons with other biopsy methods and explore comparative health economic & cost benefit analysis.

## Supplemental Material

Camprobe_JCU_Supplementary_1 – Supplemental material for Multicentre clinical evaluation of the safety and performance of a simple transperineal access system for prostate biopsies for suspected prostate cancer: The CAMbridge PROstate Biopsy DevicE (CamPROBE) study Click here for additional data file. Supplemental material, Camprobe_JCU_Supplementary_1 for Multicentre clinical evaluation of the safety and performance of a simple transperineal access system for prostate biopsies for suspected prostate cancer: The CAMbridge PROstate Biopsy DevicE (CamPROBE) study by Vincent J Gnanapragasam, Kelly Leonard, Michal Sut, Cristian Ilie, Jonathan Ord, Jacques Roux, Maria Consuelo Hart Prieto, Anne Warren and Priya Tamer in Journal of Clinical Urology

Camprobe_JCU_Supplementary_2 – Supplemental material for Multicentre clinical evaluation of the safety and performance of a simple transperineal access system for prostate biopsies for suspected prostate cancer: The CAMbridge PROstate Biopsy DevicE (CamPROBE) study Click here for additional data file. Supplemental material, Camprobe_JCU_Supplementary_2 for Multicentre clinical evaluation of the safety and performance of a simple transperineal access system for prostate biopsies for suspected prostate cancer: The CAMbridge PROstate Biopsy DevicE (CamPROBE) study by Vincent J Gnanapragasam, Kelly Leonard, Michal Sut, Cristian Ilie, Jonathan Ord, Jacques Roux, Maria Consuelo Hart Prieto, Anne Warren and Priya Tamer in Journal of Clinical Urology
