# Supplementary material for: Multicentre clinical evaluation of the safety and performance of a simple transperineal access system for prostate biopsies for suspected prostate cancer: The CAMbridge PROstate Biopsy DevicE (CamPROBE) study
Source: J Clin Urol. 2020 Jun 12;13(5):364–70. doi: 10.1177/2051415820932773 (PMC7521793; doi:10.1177/2051415820932773)
Supplement: Camprobe_JCU_Supplementary_1 – Supplemental material for Multicentre clinical evaluation of the safety and performance of a simple transperineal access system for prostate biopsies for suspected prostate cancer: The CAMbridge PROstate Biopsy DevicE (CamPROBE) study [file Camprobe_JCU_Supplementary_1.docx]

Evaluation of effects on patients undergoing CAMPROBE local anaesthetic transperineal prostate biopsies

CAMPROBE Q1

**Please write your initials here……………………………………….**

**Date of biopsy: Centre number :**

**Study number :**

**Administer immediately after biopsy**

This questionnaire is designed to help us understand how much discomfort the procedure you have just had caused you. **For each of the questions, please place a tick in the box ✓ that most accurately indicates your experience. Please tick only one box for each question.**

| **1.** | How much **discomfort** did the initial blood test cause? | | | | | | | | | | | | | | | | | |
| --- | --- | --- | --- | --- | --- | --- | --- | --- | --- | --- | --- | --- | --- | --- | --- | --- | --- | --- |
|  | | **_0_** | | **_1_** | | **_2_** | **_3_** | **_4_** | **_5_** | **_6_** | **_7_** | | **_8_** | **_9_** | | |  | |
|  | |  | |  | |  |  |  |  |  |  | |  |  | | |  | |
| **none** | | |  | | | | | | | | | | | | **a great deal** | | | |
|  |  | | | |  | | | | | | |  | | | |  | |  |

| **2.** | How much **discomfort** did the initial prostate examination (finger in the back passage) cause you? | | | | | | | | | | | | | | | | | |
| --- | --- | --- | --- | --- | --- | --- | --- | --- | --- | --- | --- | --- | --- | --- | --- | --- | --- | --- |
|  | | **_0_** | | **_1_** | | **_2_** | **_3_** | **_4_** | **_5_** | **_6_** | **_7_** | | **_8_** | **_9_** | | |  | |
|  | |  | |  | |  |  |  |  |  |  | |  |  | | |  | |
| **none** | | |  | | | | | | | | | | | | **a great deal** | | | |
|  |  | | | |  | | | | | | |  | | | |  | |  |

| **3.** | How much **discomfort** did the insertion of the scanner probe cause you? | | | | | | | | | | | | | | | | | |
| --- | --- | --- | --- | --- | --- | --- | --- | --- | --- | --- | --- | --- | --- | --- | --- | --- | --- | --- |
|  | | **_0_** | | **_1_** | | **_2_** | **_3_** | **_4_** | **_5_** | **_6_** | **_7_** | | **_8_** | **_9_** | | |  | |
|  | |  | |  | |  |  |  |  |  |  | |  |  | | |  | |
| **none** | | |  | | | | | | | | | | | | **a great deal** | | | |
|  |  | | | |  | | | | | | |  | | | |  | |  |

|  |  | | | |  | | | | | | |  | | | |  | |  |
| --- | --- | --- | --- | --- | --- | --- | --- | --- | --- | --- | --- | --- | --- | --- | --- | --- | --- | --- |
| **4.** | How **uncomfortable** was the presence of the probe in your back passage? | | | | | | | | | | | | | | | | | |
|  | | **_0_** | | **_1_** | | **_2_** | **_3_** | **_4_** | **_5_** | **_6_** | **_7_** | | **_8_** | **_9_** | | |  | |
|  | |  | |  | |  |  |  |  |  |  | |  |  | | |  | |
| **not at all** | | |  | | | | | | | | | | | | **a great deal** | | | |
|  |  | | | |  | | | | | | |  | | | |  | |  |

| **5.** | How much **discomfort** did the injection of local anaesthetic cause you? | | | | | | | | | | | | | | | | | | |
| --- | --- | --- | --- | --- | --- | --- | --- | --- | --- | --- | --- | --- | --- | --- | --- | --- | --- | --- | --- |
|  | | **_0_** | | **_1_** | | **_2_** | **_3_** | **_4_** | **_5_** | **_6_** | **_7_** | | **_8_** | **_9_** | | |  | | |
|  | |  | |  | |  |  |  |  |  |  | |  |  | | |  | | |
| **none** | | |  | | | | | | | | | | | | **a great deal** | | | | |
|  |  | | | |  | | | | | | |  | | | |  | |  |  |

| **6.** | How much **discomfort** did the actual taking of the biopsies with the needle cause you? | | | | | | | | | | | | | | | | | |
| --- | --- | --- | --- | --- | --- | --- | --- | --- | --- | --- | --- | --- | --- | --- | --- | --- | --- | --- |
|  | | **_0_** | | **_1_** | | **_2_** | **_3_** | **_4_** | **_5_** | **_6_** | **_7_** | | **_8_** | **_9_** | | |  | |
|  | |  | |  | |  |  |  |  |  |  | |  |  | | |  | |
| **none** | | |  | | | | | | | | | | | | **a great deal** | | | |
|  |  | | | |  | | | | | | |  | | | |  | |  |

**Perception Questionnaire**

This questionnaire asks about your perceptions of the biopsy you have just had. Please answer each question by placing a tick ✓ in the appropriate box. Please tick only one box for each question.

|  |  | |  | |  | |  |  |
| --- | --- | --- | --- | --- | --- | --- | --- | --- |
| **7.** | | Overall, how **painful** did you find the whole procedure? | | | | | |  |
|  |  | | | not at all | | |  | _0_ |
|  |  | | |  | |  |  |  |
|  | a little | | | | | |  | _1_ |
|  |  | | |  | |  |  |  |
|  | somewhat | | | | | |  | _2_ |
|  |  | | | | |  |  |  |
|  | a lot | | | | | |  | _3_ |
|  |  | |  | |  | |  |  |

|  |  | |  | |  | |  |  |
| --- | --- | --- | --- | --- | --- | --- | --- | --- |
| **8.** | | Overall, how **physically uncomfortable** did you find the whole procedure? | | | | | |  |
|  |  | | | not at all | | |  | _0_ |
|  |  | | |  | |  |  |  |
|  | a little | | | | | |  | _1_ |
|  |  | | |  | |  |  |  |
|  | somewhat | | | | | |  | _2_ |
|  |  | | | | |  |  |  |
|  | a lot | | | | | |  | _3_ |
|  |  | |  | |  | |  |  |

|  |  | |  | |  | |  |  |
| --- | --- | --- | --- | --- | --- | --- | --- | --- |
| **9.** | | Overall, how **embarrassing** did you find the whole procedure? | | | | | |  |
|  |  | | | not at all | | |  | _0_ |
|  |  | | |  | |  |  |  |
|  | a little | | | | | |  | _1_ |
|  |  | | |  | |  |  |  |
|  | somewhat | | | | | |  | _2_ |
|  |  | | | | |  |  |  |
|  | a lot | | | | | |  | _3_ |
|  |  | |  | |  | |  |  |

|  |  | |  | |  | |  |  |
| --- | --- | --- | --- | --- | --- | --- | --- | --- |
| **10.** | | Overall, how much **loss of dignity** did you feel? | | | | | |  |
|  |  | | | not at all | | |  | _0_ |
|  |  | | |  | |  |  |  |
|  | a little | | | | | |  | _1_ |
|  |  | | |  | |  |  |  |
|  | somewhat | | | | | |  | _2_ |
|  |  | | | | |  |  |  |
|  | a lot | | | | | |  | _3_ |
|  |  | |  | |  | |  |  |

|  |  | |  | |  | |  |  |
| --- | --- | --- | --- | --- | --- | --- | --- | --- |
| **11.** | | Overall, how much of a problem would you find having **a similar biopsy** in the future? | | | | | |  |
|  |  | | | not a problem | | |  | _0_ |
|  |  | | |  | |  |  |  |
|  | a minor problem | | | | | |  | _1_ |
|  |  | | |  | |  |  |  |
|  | a moderate problem | | | | | |  | _2_ |
|  |  | | | | |  |  |  |
|  | a major problem | | | | | |  | _3_ |
|  |  | |  | |  | |  |  |

|  |  | |  | |  | |  |  |
| --- | --- | --- | --- | --- | --- | --- | --- | --- |
| **12.** | | Overall, if you were discussing the procedure with a friend **who requires a biopsy** in the future, how would you describe it? | | | | | |  |
|  |  | | | a minor procedure | | |  | _0_ |
|  |  | | |  | |  |  |  |
|  | a moderate procedure tolerable under local anaesthetic | | | | | |  | _1_ |
|  |  | | |  | |  |  |  |
|  | quite a major procedure but tolerable under local anaesthetic | | | | | |  | _2_ |
|  |  | | | | |  |  |  |
|  | a major procedure that requires a general anaesthetic (being put to sleep) | | | | | |  | _3_ |
|  |  | |  | |  | |  |  |
