# Supplementary material for: Multicentre clinical evaluation of the safety and performance of a simple transperineal access system for prostate biopsies for suspected prostate cancer: The CAMbridge PROstate Biopsy DevicE (CamPROBE) study
Source: J Clin Urol. 2020 Jun 12;13(5):364–70. doi: 10.1177/2051415820932773 (PMC7521793; doi:10.1177/2051415820932773)
Supplement: Camprobe_JCU_Supplementary_2 – Supplemental material for Multicentre clinical evaluation of the safety and performance of a simple transperineal access system for prostate biopsies for suspected prostate cancer: The CAMbridge PROstate Biopsy DevicE (CamPROBE) study [file Camprobe_JCU_Supplementary_2.docx]

**Please circle**

**CAMPROBE study visual pain scale Date of biopsy : Centre number: Study number :**

**Administer immediately after biopsy**
